# Going Remote: Evaluating a Global Health Practicum Program During COVID-19 Travel Restrictions

**DOI:** 10.5334/aogh.3687

**Published:** 2022-10-17

**Authors:** Meagan Harrison, Eumihn Chung, Dan Kajungu, Tanmay Mahapatra, Mahbubur Rahman, Marius-Ionut Ungureanu, Anna Kalbarczyk

**Affiliations:** 1 Johns Hopkins Bloomberg School of Public Health, Johns Hopkins Center for Global Health, Baltimore, MD, US; 2 Makerere University Centre for Health and Population Research, Kampala, Uganda; 3 CARE India Solutions for Sustainable Development, Department of Concurrent Measurement and Learning, Bihar, India; 4 International Centre for Diarrhoeal Disease Research, Bangladesh (icddr,b), Division of Infectious Diseases, Dhaka, Bangladesh; 5 Babeș-Bolyai University, Department of Public Health, Cluj-Napoca, Romania

**Keywords:** Global Health Training, Global Health Practicum, STEGH, Twinning, COVID-19

## Abstract

**Background:**

Short-term experiences in global health (STEGHs) are an important part of global health degree programs. Due to the COVID-19 pandemic, travel was not possible for students planning to participate in the Johns Hopkins Center for Global Health’s Global Health Established Field Placement (GHEFP) program in 2020. Working with willing faculty mentors, in-country collaborators, and students, the Center allowed students to complete their practicums remotely so that students could gain practicum experience despite not being able to travel, and faculty and collaborators could receive the planned support on their projects.

**Objectives:**

This evaluation aims to describe the experience of pivoting the GHEFP program from an in-person, in-country program to a remote practicum.

**Methods:**

We analyzed program evaluation data from 30 students, 20 faculty members, and 10 in-country collaborators. Surveys for each group consisted of multiple choice, scale rating, and open-ended questions. The quantitative data was analyzed using Microsoft Excel to calculate survey response frequencies. The open-ended responses were analyzed for emergent themes.

**Findings:**

The remote GHEFP experience enabled students to gain practice working on global health projects from a distance, but it came with challenges related to preparation, communication, shifting scopes of work, and contextualization. All participants would have preferred an in-person experience if given a choice, but most agreed that a remote practicum was better than not participating at all.

**Conclusions:**

The remote program served its purpose during the height of the pandemic. Given the hybrid nature of global health today, many aspects of the remote practicum experience are helpful for global health training. Future iterations of remote STEGHs should initially be designed for remote work to ensure meaningful scopes for students that are helpful to faculty mentors and collaborators. Hybrid models may also be useful. Mutually beneficial twinning relationships should also be incorporated into remote and in-person STEGHs to foster a more equitable global health training environment.

## Background

Short-term experiences in global health (STEGHs) are an important part of many public health degree programs, and global health trainees rely on immersive practicums to gain experience working in different countries with international partners and organizations [1,2].

Due to the COVID-19 pandemic and consequent lockdowns and travel restrictions, traditional STEGHs were not possible for over a year. Similar to how academic institutions were forced to shift to remote asynchronous teaching and learning, the pandemic required programs to rethink their applied learning experiences [1,3]. Rather than offering in-person immersive experiences, many programs pivoted to train and engage students virtually [3]. For example, Child Family Health International (CFHI), a United Nations–recognized leader in global health education, launched a variety of virtual global health opportunities, offering webinars, online courses, virtual internships, and practica. The CHFI virtual internship and practicum program was offered in collaboration with partners in eight different countries and lasted either four, six, or eight weeks. It featured competency-informed readings, intercultural training through CFHI partner Aperian Global, virtual language school (where necessary), seminars, facilitated group reflection sessions, cultural learning and virtual immersion, and project-based work to support operations and objectives of the host organization/partners [4].

Perhaps a silver lining of the pandemic, shifting from in-person to remote training experiences presents an exciting opportunity to address certain underlying and well-established power inequities in global health. Critics of traditional STEGHs have argued that these programs run the risk of primarily benefiting the students visiting from high-income countries more than the host organization while placing a burden on the host organizations in low-income countries to facilitate a learning experience for the student’s gain; this contributes to the power imbalances that are inherent in the colonialistic and “extractive research” style that has been a hallmark of the global health research industry [5,6,7,8]. Remote training opportunities, however, provide a mechanism for globally diverse trainees who may lack the ability to travel to a training location due to cost, visa restrictions, or other reasons, to benefit from participating in a class or practicum experience virtually. As digital equity (and specifically internet access) around the world increases, the rise of remote learning opportunities improves access to and participation in these important educational and training experiences [3,9].

In response to the impact that COVID-19 had on global travel, the Johns Hopkins Center for Global Health (CGH) transitioned its 2020–2021 Global Health Established Field Placement (GHEFP) program to a fully virtual format for the first time in program history. This program, launched over a decade ago, provides $3,500 travel grants to Johns Hopkins students to work with faculty members on their research and practice projects in low- and middle-income countries (LMICs). GHEFP was designed to provide resources for students to work with global health mentors and attain international cross-cultural field experience. While this has traditionally required a commitment from students to spend at least six weeks in-country, we pivoted to support applied learning for students in a virtual environment.

In this paper, we share the experience of implementing the remote GHEFP program during the pandemic and share feedback we received from students, faculty, and in-country collaborators upon completion of the experience.

## Methods

### Virtual program pivot

Forty-four faculty projects were matched with students for the 2020–2021 GHEFP cycle. When it became clear that the COVID-19 pandemic would be a sustained interruption to pre-pandemic life and travel conventions and that student travel would not be possible, the CGH began pivoting the GHEFP program to a remote format.

We reached out to students, faculty, and in-country collaborators to offer them the opportunity to develop meaningful remote scopes of work for the students. If they were interested in doing so, they worked together to submit a revised version of their previously submitted Faculty-Student-Collaborator Agreements, which indicated the buy-in of all three parties to engage in the GHEFP together. We required that the revised agreements outline the proposed remote scope of work and detailed communication plan during the proposed time of remote student engagement.

All 44 projects were offered the opportunity, and 30 sites chose to submit plans to engage together remotely. Upon review, the CGH approved all 30 revised scopes of work. CGH dispersed $2,000 awards to support student living expenses while working on their projects, a reduced amount compared to the original award because they no longer needed to pay for travel costs.

### Program reporting requirements and evaluation

Upon completion of their remote practicums, CGH required all students to create a poster and display it during a poster session at Global Health Day, an annual symposium hosted by CGH. Global Health Day 2021 and the poster session were held virtually.

Students were also required to complete an evaluation survey of their experience. The surveys asked 37 multiple choice or rating scale questions on student preparation for the placement, the student’s experience with the CGH during the award process, and the effect of their participation in the GHEFP program on student learning objectives. It also included open-ended questions to capture qualitative comments on their experiences. The questions were adapted from past program surveys to reflect the remote nature of the practicum where necessary, and specific questions were designed to explore the remote practicum experience specifically. The survey was designed in Qualtrics^©^, an online survey software, and distributed via email to all students and program staff.

CGH also requested, but did not require, faculty mentors and in-country site collaborators to complete a survey on their experience hosting a student working remotely on their project. The faculty mentor and in-country site collaborator surveys included 32 and 50 multiple choice, scale rating, and open-ended questions, respectively. CGH program staff emailed Qualtrics survey links to faculty and collaborators and followed up via email.

### Analysis

The quantitative data were analyzed using Microsoft Excel to calculate survey response frequencies. The qualitative data from the open-ended responses were analyzed by categorizing responses to identify emergent themes and were then summarized.

This secondary data analysis was approved by the Johns Hopkins Bloomberg School of Public Health Institutional Review Board. No consent was required.

## Findings

When presented with the opportunity, 68% (n = 30) of the 44 students awarded GHEFPs in the year 2020 chose to participate in the remote placement option with their sites during the one-year award cycle. All (100%, n = 30) students who participated in a remote placement completed the evaluation survey. The response rate for the 22 faculty mentors (some faculty mentors hosted more than one site and student) was 91% (n = 20). Only 34% (n = 10) of the 29 site collaborators responded to the post-program survey (Table 1).

**Table 1:** Student demographic and placement information (n = 30).


VARIABLES	n (%)

Student status	

Graduate	28 (93.3)

Undergraduate	2 (6.7)

Degree pursuing	

MSPH	17 (56.7)

PhD	5 (16.7)

MHS	3 (10.00)

Bachelor’s	2 (6.7)

MS	2 (6.7)

MSN	1 (3.3)

School/Department affiliation	

School of Public Health	14 (46.7)

International Health	6 (20.0)

Population, Family and Reproductive Health	3 (10.0)

Epidemiology	3 (10.0)

School of Nursing	2 (6.7)

School of Arts and Sciences (*Public Health Studies*)	2 (6.7)

School of Engineering (*Biomedical Engineering*)	

Reasons for participating in GHEFP*	

To gain helpful skills	19 (63.3)

As part of capstone or dissertation work	12 (40.0)

To go into a career in global health	12 (40.0)

To explore if a career in global health is a good fit	10 (33.3)

To complete a practicum for a degree requirement	10 (33.3)


* Students could select more than one option.

Of the 30 students who participated in the remote GHEFP program, 33.3% (n = 10) completed their placement for a degree requirement, and 40% (n = 12) participated as part of their capstone or dissertation work. Other reasons for participating included a desire to gain helpful skills (63.3%, n = 19), a desire to pursue a career in global health and wanting to get more experience (40%, n = 12), and participating to explore if a career in global health may be a good fit (33.3%, n = 10). Fifty percent (n = 15) of the placement projects were in Africa, 33.3% (n = 10) were in Asia, 13.3% (n = 4) were in Latin America, and 3% (n = 1) were in Europe.

**Figure 1:**
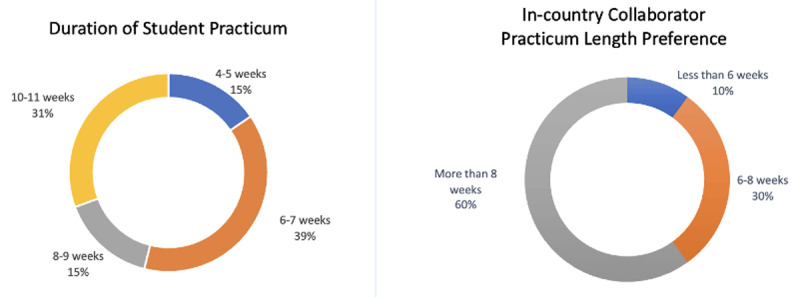
Duration of student practicums and in-country collaborator practicum length preferences.

By the end of the GHEFP cycle, 56.7% (n = 17) of awardees spent 12 or more weeks working on their placements. Roughly 13% (n = 4) spent 10–11 weeks, 6.7% (n = 2) spent 8–9 weeks, 16.7% (n = 5) spent 6–7 weeks, and 6.7% (n = 2) spent 4–5 weeks working on their placements (Figure 1). In-country collaborators were asked about the length of time they prefer to work with students on short-term placements, and 60% (n = 6) preferred more than 8 weeks, 30% (n = 3) preferred 6–8 weeks, and 10% (n = 1) preferred less than 6 weeks.

During their virtual placements, 80% (n = 24) of students met with their project team members 1–2 times per week, and 10% (n = 3) met 3–4 times per week. Ten percent (n = 3) indicated that they never met with their in-country counterparts.

### Preparation for the remote GHEFP

All faculty members who responded to the survey indicated that they had a discussion and agreement about the scope of work the students would assume prior to beginning the placement. Nearly all students (93.3%, n = 28) responded that a discussion of remote participation expectations occurred with their faculty mentor or in-country collaborators. Ninety percent (n = 9) of the in-country collaborators discussed both remote communication plans and scope of work and responsibilities with their students. Over half the in-country collaborators (60%, n = 6) reported that they discussed and provided information about navigating cultural experiences, logistics, differences, and/or challenges with their students.

When asked about their overall preparation for their remote practicum experience, 70% (n = 21) of GHEFP students responded, “I was as well prepared as I could have been,” and 23.3% (n = 7) answered, “I was fairly well prepared but could have avoided a few problems with a bit more information.” In the open-ended responses, some students wished they would have read more literature on their topics before engaging with the work. Others noted that they would have benefited from “learning about the cultural working styles” before engaging remotely with their teams. A handful of students shared that they could have been more prepared for the remote practicum experience if there had been a more contextual orientation to their specific projects, as they were not able to be there in-person. One student shared,

There was so much information about travel, safety, etc., but not as much content as to how to operate day-to-day in a research context, and especially remotely. I really enjoyed my field placement, but I think some extra preparation in that regard would have been nice, regardless of traveling to the country or not.

### Student experience

Overall, student participants responded that the remote experience was helpful for their understanding of a variety of topics. Forty-three percent (n = 13) of students said that the experience was excellent for helping their understanding of public health issues affecting the people in the countries in which they worked, and 33.3% (n = 10) indicated that the remote experience was excellent for helping them to understand the health systems of the country. Only 10% (n = 3) responded that the experience excellently helped them to understand the daily life of people in the country (Table 2).

**Table 2:** Usefulness of remote experience to students for understanding key topics (n = 30).


	EXCELLENT n (%)	GOOD n (%)	FAIR n (%)	POOR n (%)	N/A n (%)

Public health issues affecting the people in the country with which you worked	13 (43.3)	11 (36.7)	3 (10)	2 (6.7)	1 (3.3)

Health systems of the country	10 (33.3)	9 (30)	7 (23.3)	2 (6.7)	2 (6.7)

Differences in clinical care experienced in developing countries	11 (36.7)	9 (30)	3 (10)	3 (10)	4 (13.3)

Stakeholders and their interests in your project	9 (30)	10 (33.3)	4 (13.3)	5 (16.7)	2 (6.7)

How to conduct research	16 (53.3)	5 (16.7)	6 (20)	2 (6.7)	1 (3.3)

Nuances of conducting research/practice work in resource-poor settings	12 (40)	9 (30)	4 (13.3)	3 (10)	2 (6.67)

Daily life of people in the country	3 (10)	9 (30)	7 (23.3)	6 (20)	5 (16.7)


Students were also asked to compare their global health skills and abilities before and after their placements. Sixty percent (n = 18) of students indicated that they were more able to apply relevant scientific research methods in different contexts than before their remote placement. Sixty percent (n = 18) indicated they were more able to analyze complex global health challenges after the placement compared to before. When asked if they were more able to develop solutions in response to complex global health challenges, 56.7% (n = 17) indicated they were more able than before the placement (Table 3).

**Table 3:** Student perceptions of skills and abilities after the remote international experience (n = 30).


	MORE ABLE THAN BEFORE THE PLACEMENT N (%)	THE SAME ABILITY AS BEFORE THE PLACEMENT N (%)	LESS ABLE THAN BEFORE THE PLACEMENT N (%)

Ability to apply relevant scientific research method(s) in different contexts?	18 (60.0)	11 (36.7)	1 (3.3)

Ability to analyze complex global health challenges?	18 (60.0)	11 (36.7)	1 (3.3)

Ability to develop solutions in response to complex global health challenges?	17 (56.7)	12 (40.0)	1 (3.3)


When asked if they experienced culture shock during the remote placement, 96.7% (n = 29) responded no, and 3.3% (n = 1) replied yes. In the qualitative data, one respondent shared that differences in work style and culture were prominent in their placement and that it would have been helpful to have more of an orientation in that regard.

During their placement, 80% (n = 24) of students felt very valued by their faculty supervisor/mentors, 13.3% (n = 4) felt somewhat valued, and 6.7% (n = 2) did not feel valued at all. Seventy percent (n = 21) of students felt very valued by their in-country placement supervisors/mentors, 20% (n = 6) felt somewhat valued, and 10% (n = 3) did not feel valued at all.

In the qualitative responses, many students shared stories of excellent mentorship from both their faculty mentors and in-country collaborators. One student shared,

[My faculty mentor] has been a great support. He never let our virtual working challenges come in the way of accomplishing the tasks at hand. … He set realistic expectations and was very understanding of the challenges of working on this project remotely from a different time zone… . He opened a channel for close and non-hesitant communication, which was very helpful especially during a virtual working environment.

Another student shared how their mentor sponsored opportunities beyond the GHEFP program, inviting the student to be a teaching assistant for a class and helping them to expand their network within the project team and beyond. “He always created an environment of inclusivity for me,” said the student.

### Student sentiments

Students indicated that they did encounter difficulties during the GHEFP experience. A few expressed challenges with delays on projects that subsequently changed the available scope of work compared to what was originally agreed upon. For one student, this led to feelings of not being needed by their faculty and in-country team:

Obviously COVID-19 changed everything, but the remote experience was just not really what I signed up for. I felt like I couldn’t contribute anything, and they didn’t need me on the study. I was hoping to do analysis, but I mostly did administrative work, as everything was so delayed. I definitely would have had a much better experience without COVID.

Others shared that limited interaction time with the in-country teams and time zone differences proved challenging. “It was hard to coordinate data collection without having much one-on-one time with the project team, who were in a completely different time zone,” shared one student. “I got incredible support from my team, however technical constraints drastically slowed down the process for myself and my team.” They also expressed that not all projects transition well to remote work.

Despite the challenges, in the qualitative responses, most students expressed gratitude to have an opportunity to work on a practicum at all during the pandemic and gave high praise for the GHEFP program’s transition to support remote practicums. “Although I did not get to go abroad, I was able to establish a lot of connections with my current project and plan for future opportunities,” shared another GHEFP student. The students also expressed an understanding that everyone involved in the GHEFP program (faculty mentors, in-country mentors, and the students themselves) was doing their best under new and unprecedented circumstances.

Many students wished to continue working with their teams even after the GHEFP ended. Two-thirds (n = 20) of students indicated that at the time of the survey, they were developing or had developed a plan for continued engagement in the project/placement. One student shared,

I would love to continue working on the project alongside my mentor. The guidance and support are unparalleled and we worked great together. I am in a stage of career building in global public health and this type of mentorship can be very helpful for me in career development and learning.

### Faculty sentiments

In their qualitative responses, faculty members also shared varied experiences. Some faced challenges related to the remote work format. One faculty member shared,

While both students worked hard at tasks I assigned them during the remote internship, not being able to travel and participate directly in the field work was frustrating for them and for me. There was data collection we were unable to do because the students could not be there to do it. And frankly, it was hard to find meaningful work for them to do remotely.

Some responses were extremely positive. As one faculty member described,

We had amazing students this year and they contributed substantially despite the 2020 challenges… . We, in fact, have hired one of them full-time for this coming year, and another is also being hired full-time… . Overall, our students have been such a delight and the quality of their engagement with the projects was amazing, even without visiting/spending time at the sites.

Many responses fell somewhere in the middle. “I had two students work with me this year,” shared one faculty member. “They were both very nice, eager to work and help, but the remote working made it much harder to engage with them relative to them being in-country with me.” Another faculty member said,

This year was different and challenging which is also a good training for the students. The student was supposed to visit the field for capacity building. Instead, she adapted the training materials for online training and performed the training. She understood the problems and how to mitigate them. It was a unique experience for all of us.

Nineteen of the 20 faculty respondents answered questions regarding their perspective comparing the remote experience versus the in-person experience of hosting students to work on their projects (Table 4).

**Table 4:** Faculty and collaborator perspectives on remote vs. in-person experience.


	BETTER REMOTE, n (%)		BETTER IN-PERSON, n (%)		NO DIFFERENCE, n (%)	
		
	FACULTY (n = 19)	COLLABORATOR (n = 10)	FACULTY (n = 19)	COLLABORATOR (n = 10)	FACULTY (N = 19)	COLLABORATOR (n = 10)

Overall benefit to project of engaging a student	0 (0)	1 (10)	13 (68.4)	8 (80)	6 (31.2)	1 (10)

Communication with student	2 (10.5)	2 (20)	8 (42.1)	6 (60)	9 (47.4)	2 (20)

Experience of in-country team working with student	0 (0)	–	17 (89.5)	–	2 (10.5)	–

Which do you prefer? Hosting a remote student or hosting an in-person student?	0 (0)	2 (20)	14 (73.7)	7 (70)	5 (26.3)	1 (10)


Sixty-eight percent (n = 13) of faculty respondents indicated that the overall benefit to the project of engaging a student was better during in-person engagement versus 31.2% (n = 6) who responded there was not a difference, and none felt that the overall benefit was better with the remote format. Regarding communication between the student and faculty member, 10.5% (n = 2) said it was better remotely, 42.1% (n = 8) said it was better in-person, and 47.4% (n = 9) mentioned they found no difference. Roughly 90% (89.5%, n = 17) of faculty members indicated that the experience of the in-country team was better in-person compared to 10.5% (n = 2) who indicated no difference, while no faculty members reported a better experience for their in-country team with a remote student. Finally, when asked if they prefer hosting a student remotely versus in-person, no faculty members preferred the remote format over the in-person format, while 73.7% (n = 14) preferred in-person and 26.3% (n = 5) did not prefer one over the other.

### Collaborators’ experience

Eighty percent (n = 8) of the in-country collaborators said that their students were engaged or very engaged in the projects, while 20% (n = 2) described their students as somewhat engaged or rarely engaged. Fifty percent (n = 5) of in-country collaborators said that their students became more engaged over time, while 30 (n = 3) remained engaged at consistent levels throughout.

Fifty percent (n = 5) of collaborators agreed that having a GHEFP student work with them was overall beneficial to the project, 30% (n = 3) neither agreed nor disagreed, and 20% (n = 2) disagreed. Forty percent (n = 4) of collaborators agreed that the benefit of having a student at the placement outweighed the time and effort of training and supervision, while 10% (n = 1) disagreed. All collaborators agreed, however, that they would like to participate in the program again.

Eighty percent (n = 8) of collaborators said that the overall benefit of hosting a student to the project would be better in-person, 10% (n = 1) indicated remote was better, and 10% (n = 1) indicated there would be no difference to the benefit whether a student was remote or in-person. Sixty percent (n = 6) indicated that communication with the student was better in-person, 20% (n = 2) said it was better remotely, and 20% (n = 2) indicated no difference. Seventy percent (n = 7) of the collaborators preferred hosting a student in-person, while 20% (n = 2) preferred hosting a student remotely (Table 4). When reflecting on the experience, one collaborator shared, “I have successfully mentored many students in the program, but this year it was not smooth due to COVID-19. I very much look forward to the next student mentoring opportunity.”

### Discussion

Overall, the remote GHEFP experience served its purpose of enabling students to gain practice working on global health projects, but it came with common drawbacks that led to varied feedback from those who participated. An in-person experience would have been preferred by all if given the choice.

From the student perspective, results from the evaluation demonstrated that their remote experience was useful for understanding most key topics except for understanding more about the daily life of people in-country, which was expected without being able to travel for an immersive experience. The evaluation also demonstrated an increase of self-perceived skills and abilities in most students for various global health competencies. Additionally, two-thirds of students indicated that they were planning to continue working with their teams on the project after the GHEFP experience ended, and at least two have been hired full time by their faculty mentors after graduation, all tangible outcomes of positive practicum collaborations.

From the faculty mentor perspective, 85% chose to continue to be engaged with their students after they finished their placement. Many shared positive anecdotes about having the student work with them and their teams. Some even shared that communication with their students was better with them working remotely versus in-country, which may be explained by more time spent online or better access to the internet compared to when the students are working in LMICs. Most in-country collaborators shared that having a student work with them was beneficial to their projects overall, and all would like to participate in the program again as collaborators. Most faculty and in-country collaborators agreed, however, that they prefer hosting students in-person.

One interesting finding was that the students and faculty differed in their responses on their views on preparation for the GHEFP placement. While all faculty members shared that they provided training, some students felt that they needed more or different preparation. The divergence of opinion on whether adequate training was provided should be explored. The literature frequently includes inadequate preparation as a critique of STEGHs, citing variable degrees of depth of training and a focus on participant safety and objectives rather than on responsibility, community impacts, and hard skills [10,11]. The CGH places great importance on preparing students for traveling for the GHEFP and developed a travel preparation course that includes broader competencies for living and working in a different country. A systematic review on predeparture preparation resources conducted by the CGH encouraged early engagement with international partners, the inclusion of site-specific content during preparation, and utilization of interactive approaches to learning [12]. Because each GHEFP scope of work is unique, however, the respective faculty mentors, not CGH, are responsible for providing orientations to their specific projects and any on-the-job training that a student might need to complete their scope of work. While it is not feasible for CGH itself to provide specific training for each individual project and scope of work, there may be a need for CGH to add more structured requirements for the way faculty mentors conduct orientations for the students in both the technical aspects of their projects and the work/organizational culture of their projects in addition to cultural and societal norms. Prior and continuous engagement with collaborating sites may better equip students, faculty, and in-country collaborators to work with each other.

While the overall experience was positive, certain drawbacks came with not being able to participate in-country. For some students, depending on the nature of their tasks, limited interaction with the in-country teams and time zone differences made progress on the work slower, even with good support from the teams. There were also significant COVID-19 delays with some projects that changed the original scope of work, as faculty members and in-country collaborators were faced with restrictions due to lockdowns and other pandemic-related challenges. Understandably, this led to unmet expectations on the part of some students, who experienced lack of clarity in their role due to the shifting needs of the project, sometimes leaving them without significant work. Faculty members and collaborators experienced similar frustrations, finding it challenging to create meaningful work when the students could not travel to the field. As is the nature of a global pandemic, the disruption of global health operations, including rapidly shifting priorities, personal safety concerns, communication challenges, and limited in-person activities, was not unique to our projects and partnerships; everyone was affected in some way [13,14].

Some of the challenges may have been due to the remote work setup, but it was difficult to parse out what was problematic because of the distance and what was challenging because of delays related to COVID-19. Even before the pandemic, telecommuting has been on the rise, bringing increased job satisfaction and productivity along with it [15], and the global health community is especially accustomed to collaborating with partners remotely, across time zones and country borders. In a nonpandemic environment, where project delays were fewer, the remote experience may have had fewer challenges. Most of the challenges described in this evaluation seemed to stem more from the effects of the pandemic restrictions and delays than the nature of remote work in general.

Another factor to consider is that these experiences were originally designed for in-person engagement but were subsequently adapted to a remote format after the fact, and some scopes of work translate better to remote work than others. In the future, it could be helpful to explore whether placements that are specifically designed for remote work initially might produce better experiences.

Remote or in-person, we propose that one way to foster more meaningful experiences is to incorporate twinning into the GHEFP program by pairing GHEFP students with trainees from the host countries to work on the same project. Peer-to-peer twinning relationships would provide a meaningful, mutual, cultural exchange experience for both the student and the local twin [16,17]. This model could solve the challenge of providing more relevant cultural orientations and would allow both twins a venue to learn and teach each other about their respective work cultures, especially during a remote practicum in which interpersonal interactions are significantly more limited. Both twins could benefit from participating in the pre-practicum training and gain their own practicum experiences working on and being mentored by a Hopkins-sponsored project. This, we argue, would be a more inclusive approach to global health training than the current models employed by the GHEFP and other traditional STEGH programs.

We recognize that this evaluation does have certain limitations, including a low survey response rate from the international collaborators and limited open-ended responses from those who did respond. A possible reason for the lack of response was the increased workload for in-country collaborators during the pandemic.

## Conclusions

Overall, the remote format GHEFP served its purpose during the height of the pandemic. Given the hybrid nature of global health work now, often involving both work from a distance and travel to the field, there are aspects of the remote applied learning experience that make sense for global health training. Navigating time zone differences, asynchronous work idiosyncrasies, and cultural differences is part of the profession. Future iterations of remote STEGHs, however, should be specifically designed for remote work from the start to ensure scopes of work that are both meaningful for the students and helpful to the work of the faculty mentor and in-country collaborators and that communication and other expectations are aligned. If possible, peer-to-peer twinning relationships should be incorporated into both remote and in-person STEGHS to foster a more inclusive and equitable global health training environment.
